# Comparative evaluation of the remineralisation potential of biomimetic agents on phosphorylated dentine using SEM–EDX analysis: an in vitro study

**DOI:** 10.2340/biid.v13.45797

**Published:** 2026-04-22

**Authors:** Maruthi Gupta, Annapoorna B. M., Pravallika Chaluvadi, Rama Laxmi Koruprolu, Nikita Sebastian, Ram Sateesh Babu Mynam

**Affiliations:** a Department of Conservative Dentistry and Endodontics, Vishnu Dental College, Bhimavaram, India; b Department of Conservative Dentistry and Endodontics, JSS Dental College and Hospital, Mysuru, India; c Department of Prosthodontics, Vishnu Dental College, Bhimavaram, India; d Department of Public Health Dentistry, Vishnu Dental College, Bhimavaram, India; e Department of Conservative Dentistry and Endodontics, Christian Dental College, Ludhiana, India; f Department of Conservative Dentistry and Endodontics, Anil Neerukonda Institute of Dental Sciences, Vishakapatnam, India

**Keywords:** dentine remineralisation, biomimetic materials, sodium tripolyphosphate, CPP-ACP, theobromine, Novamin, SEM–EDX

## Abstract

**Introduction:**

Dentine remineralisation remains a clinical challenge due to its higher critical pH and complex organic matrix. Biomimetic strategies that replicate the role of non-collagenous proteins through phosphorylation have shown promise in promoting intrafibrillar mineralisation. Sodium tripolyphosphate (STPP) has been proposed as a phosphorylating agent capable of enhancing dentine remineralisation. This in vitro study aimed to comparatively evaluate the remineralisation potential of different biomimetic agents on artificially demineralised dentine using scanning electron microscopy (SEM) and energy-dispersive X-ray (EDX) analysis.

**Methodology:**

An in vitro study was conducted using 70 extracted human permanent teeth, which were sectioned to obtain coronal dentine discs. Baseline mineral content and surface morphology were assessed using SEM and EDX. Samples were subjected to pH cycling to induce demineralisation and randomly assigned to four groups: Group A – theobromine, Group B – Casein phosphopeptide–amorphous calcium phosphate (CPP-ACP), Group C – Novamin and Group D – Artificial saliva. Each group was further subdivided based on STPP application (*n* = 10). Remineralisation protocols were carried out for 7 and 21 days, followed by SEM and EDX evaluation.

**Results:**

All specimens demonstrated significant mineral loss following demineralisation. Experimental groups showed progressive remineralisation over time compared to the control. STPP-treated subgroups exhibited greater mineral deposition and improved surface morphology. Among the tested agents, CPP-ACP combined with STPP demonstrated the highest calcium and phosphorus content, with EDX values exceeding baseline reference levels at 21 days. CPP-ACP + STPP showed significantly higher remineralisation compared to Novamin, theobromine, and the control groups at days 7 and 21 (*p* < 0.05).

**Conclusion:**

Phosphorylation of dentine using STPP enhances the remineralisation efficacy of biomimetic agents. CPP-ACP in combination with STPP showed superior remineralisation potential. These findings support the use of phosphorylating biomaterials as promising strategies for dentine remineralisation. However, the findings are limited by the in vitro design and require clinical validation.

## Introduction

Dental caries remains one of the most prevalent chronic diseases affecting both permanent and deciduous dentitions and continues to be the leading cause of irreversible tooth structure loss worldwide [1]. The disease process is dynamic in nature and is governed by a continuous balance between demineralisation and remineralisation, largely influenced by fluctuations in oral pH [2]. When acidic challenges exceed protective and reparative mechanisms, mineral loss from dental hard tissues occurs, eventually progressing to cavitation.

Enamel and dentine differ significantly in their composition, structure and response to acidic insults. Enamel consists of approximately 96% inorganic hydroxyapatite with minimal organic content, whereas dentine contains about 70% inorganic material, 20% organic matrix – predominantly type I collagen – and 10% water [3]. Due to its higher organic content and lower mineral density, dentine is more vulnerable to demineralisation. Moreover, the critical pH of dentine ranges from 6.0 to 6.9, which is substantially higher than that of enamel, making dentine more susceptible to mineral loss even under mildly acidic conditions [4].

Demineralisation is defined as the dissolution of mineral ions from tooth structure, while remineralisation refers to the redeposition of calcium and phosphate ions, restoring structural integrity and enhancing resistance to further acid attack [5]. Although enamel remineralisation has been extensively studied and successfully achieved using various therapeutic agents, dentine remineralisation remains a greater clinical challenge due to its complex ultrastructure and organic framework [6].

In dentine, demineralisation is further complicated by enzymatic degradation of the organic matrix. Acidic conditions activate endogenous matrix metalloproteinases and cysteine cathepsins, leading to degradation of exposed collagen fibrils and destabilisation of hydroxyapatite crystals [7]. In non-carious conditions such as erosion and abrasion, exposed dentine undergoes rapid mineral loss, increasing susceptibility to dentinal caries, hypersensitivity and eventual pulpal involvement [8–10]. Extensive dentine loss compromises restorative procedures and negatively affects long-term treatment outcomes.

Clinically, exposed dentine is commonly associated with conditions such as dentine hypersensitivity and non-carious cervical lesions, where increased dentinal tubule patency and mineral loss play a critical role in symptom development. Effective dentinal tubule occlusion and remineralisation are therefore essential therapeutic goals to reduce fluid movement within tubules and restore dentine integrity. The mechanism of remineralisation in enamel relies on epitaxial crystal growth on residual hydroxyapatite crystallites. These remaining crystals act as nucleation sites that attract calcium and phosphate ions, allowing crystal growth with preserved orientation and improved acid resistance [11, 12]. However, this mechanism is ineffective in dentine, as demineralised dentine often lacks residual mineral crystallites and presents an exposed collagen matrix unsuitable for conventional crystal growth [13].

Early attempts at dentine remineralisation relied on classical ion-based crystallisation approaches, which demonstrated limited success due to the absence of apatite seed crystals and collapse of the collagen scaffold [14, 15]. These limitations led to the development of biomimetic remineralisation strategies that aim to replicate the natural process of dentinogenesis.

Biomimetic remineralisation follows a non-classical, particle-mediated pathway often referred to as the ‘bottom-up’ approach [16]. In this process, liquid-like amorphous calcium phosphate (ACP) nanoprecursors infiltrate the intrafibrillar spaces of collagen and subsequently transform into hydroxyapatite, thereby restoring the hierarchical structure of dentine [17]. This strategy does not rely on pre-existing mineral crystals and is therefore more suitable for demineralised dentine.

Non-collagenous proteins (NCPs) play a critical role in physiological dentine mineralisation. Proteins such as dentine phosphoprotein and dentine matrix protein-1 contain phosphorylated residues that exhibit high affinity for calcium ions and regulate nucleation and growth of hydroxyapatite crystals [18]. However, the extraction and clinical application of NCPs are impractical due to their complexity and cost. Consequently, research has focused on identifying biomimetic analogues capable of replicating their function.

Inorganic polyphosphates have emerged as promising substitutes for NCPs. Compounds such as sodium trimetaphosphate and sodium tripolyphosphate (STPP) have demonstrated the ability to chemically phosphorylate type I collagen under alkaline conditions [19–21]. Phosphate groups from these agents can bind to demineralised collagen matrices, creating nucleation sites that facilitate intrafibrillar mineral deposition and guide biomimetic apatite formation [22, 23].

Several remineralising agents are currently available for clinical use. Casein phosphopeptide–amorphous calcium phosphate (CPP–ACP) is a well-established remineralising system that stabilises calcium and phosphate ions in an amorphous state, maintaining supersaturation and promoting mineral deposition [24]. Studies have reported enhanced dentine remineralisation when CPP–ACP is combined with phosphorylating agents, suggesting a synergistic effect [25].

Novamin, a bioactive glass composed of amorphous calcium sodium phosphosilicate, releases sodium ions upon contact with saliva, raising local pH and sustaining the release of calcium and phosphate ions [26]. These ions precipitate as hydroxycarbonate apatite, which is chemically similar to biological hydroxyapatite and has demonstrated the ability to occlude dentinal tubules and reduce hypersensitivity [27].

Theobromine (3,7-dimethylxanthine), a naturally occurring alkaloid derived from cacao, has recently gained attention as a novel remineralising agent. Previous studies have shown that theobromine can promote the formation of larger and more stable hydroxyapatite crystals in enamel, enhancing resistance to acid dissolution [28, 29]. However, limited evidence exists regarding its effect on dentine remineralisation, particularly when used in conjunction with biomimetic phosphorylation strategies.

Scanning electron microscopy (SEM) is a well-established analytical tool used to evaluate surface morphology and dentinal tubule changes following demineralisation and remineralisation [30]. When combined with energy-dispersive X-ray (EDX) spectroscopy, it enables a quantitative assessment of elemental composition, particularly calcium and phosphorus content, providing comprehensive insight into dentine remineralisation [31].

Although individual remineralising agents such as CPP-ACP, Novamin, and theobromine have been studied extensively, evidence regarding their combined use with phosphorylation strategies such as STPP remains limited, particularly on dentine substrates. So, the present study aimed to comparatively evaluate the remineralisation potential of different remineralising agents – theobromine, CPP–ACP and Novamin – on artificially demineralised dentine, with and without chemical phosphorylation using STPP, using SEM and EDX analysis.

## Methodology

### Sample selection and preparation

An in vitro study was done on a total of 70 extracted human permanent teeth, free from caries, cracks, restorations and developmental defects, which were collected following predefined inclusion and exclusion criteria. Soft tissue remnants were removed, and the teeth were cleaned using 5.25% sodium hypochlorite. Disinfection was performed by storing the samples in a 0.1% thymol solution, after which they were stored in distilled water until the initiation of the study.

### Ethical approval

Ethical approval for the use of extracted human teeth was obtained from the Institutional Ethics Committee of Vishnu Dental College (IEC No: JSSDCH IEC 42/2020). Extracted teeth were collected following informed consent and used in accordance with institutional guidelines.

### Tooth storage protocol

Extracted teeth were disinfected in 0.1% thymol solution for 24 hours and subsequently stored in distilled water at 4°C until use, for a period not exceeding 1 month, to prevent dehydration and microbial growth while preserving dentine properties.

### Preparation of dentine discs

The enamel portion of each tooth was removed using a diamond disc (Dentsply) under copious irrigation with deionised water. Coronal dentine discs were prepared and sequentially polished using fine-grit silicon carbide papers of 180, 400, 800 and 1200 grit to obtain smooth, standardised dentine surfaces. The specimens were thoroughly rinsed with deionised water to eliminate debris and stored in distilled water until mounting.

### Mounting of specimens

Silicone moulds were used to fabricate acrylic blocks using cold-cure acrylic resin. A rectangular piece of modelling wax was positioned at the centre of the mould during pouring to create a hollow space. Each dentine disc was placed into this space such that one surface remained exposed, while the opposite surface was embedded in acrylic resin. This arrangement ensured standardised exposure of the dentine surface for analysis.

### Baseline SEM and EDX analysis

Before mounting, all dentine specimens were rinsed with distilled water, air-dried on tissue paper to eliminate surface moisture and carefully handled using forceps. The samples were placed on specimen holders and evaluated under SEM at magnifications of 1000×, 3000× and 6000× to assess surface morphology. Simultaneously, EDX spectroscopy was performed to obtain baseline elemental composition values, particularly calcium and phosphorus levels.

### pH cycling

The pH-cycling protocol employed two solutions with defined compositions and conditions. The demineralising solution contained 2.2 mmol/L CaCl_2_, 2.2 mmol/L NaH_2_PO_4_ and 50 mmol/L acetic acid, adjusted to a pH of 4.8, and specimens were immersed for 8 hours per day. The remineralising solution comprised 1.5 mmol/L CaCl_2_, 0.9 mmol/L NaH_2_PO_4_ and 0.15 mol/L KCl, maintained at a pH of 7.0, with specimens immersed for 16 hours per day. This alternating immersion regimen was continued for a total duration of 14 days [5, 27].

Sample size was determined based on previous in vitro dentine remineralisation studies using SEM–EDX analysis, where comparable group sizes were shown to detect meaningful differences in mineral deposition, with the effect size of 0.412, power of 80% and alpha error of 5%, so the calculated effect size was 70 [27].

### Grouping of samples

After demineralisation, all 70 samples were randomly allocated into four main groups:

- Group A: Theobromine
- Group B: CPP-ACP
- Group C: Novamin
- Group D: Artificial saliva (Control)

Groups A, B and C were further subdivided into two subgroups (*n* = 10 per subgroup):

- Subgroup 1: Without STPP pretreatment
- Subgroup 2: With STPP pretreatment

**Figure 1:**
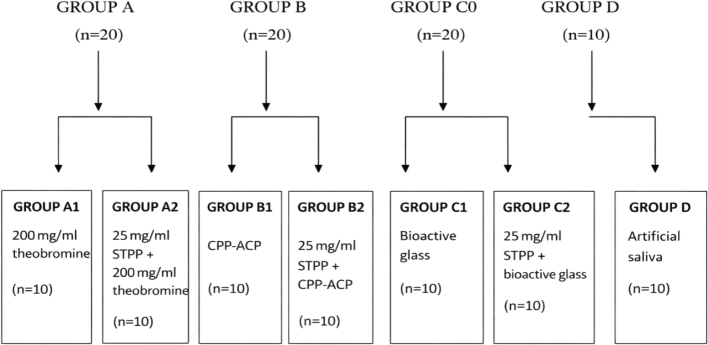
Group-wise allocation of the samples.

Group D consisted of 10 samples immersed only in artificial saliva (Figure 1).

### Remineralisation protocol

All samples were air dried for 3 seconds using oil-free compressed air before treatment.

- Subgroups A1, B1 and C1 (without STPP):
  Group A1: Theobromine solution applied using a micro brush for 3 minutes
  Group B1: CPP-ACP applied using a micro brush for 3 minutes
  Group C1: Bioactive glass (Novamin) applied using a micro brush for 3 minutes

After application, specimens were stored in 20 mL of artificial saliva. This procedure was performed twice daily, with the storage medium replaced each time.

- Subgroups A2, B2 and C2 (with STPP): Samples were pretreated with 25 mg/mL STPP for 24 hours. Following pretreatment, remineralising agents (theobromine, CPP-ACP or Novamin) were applied for 3 minutes twice daily. After each application, specimens were incubated in 20 mL of artificial saliva, which was refreshed twice daily.
- Group D (Control): Demineralised samples were stored in 20 mL of artificial saliva, with the solution replaced every 24 hours. Artificial saliva was freshly prepared daily and used for all samples.

### Evaluation period

The remineralisation protocol was carried out for 7 and 21 days. On the 7th day, samples from each subgroup (*n* = 10) were removed, rinsed with deionised water and subjected to SEM and EDX analysis. The procedure was continued for an additional 14 days, completing a 21-day cycle, after which all samples were again evaluated using SEM and EDX. The obtained values were recorded and compared with baseline measurements. SEM–EDX analysis was performed by a single calibrated examiner blinded to group allocation. Intra-examiner reliability was assessed before analysis, and the Kappa value of 0.86 indicates perfect agreement between measurements.

### Statistical analysis

Statistical analysis was performed using SPSS software version 26.0 (IBM, Chicago, USA). Quantitative data were expressed as mean ± standard deviation. One-way analysis of variance (ANOVA), followed by post hoc tests, was used to determine statistically significant differences among the groups. The level of statistical significance was set at *p* ≤ 0.05.

## Results

Baseline SEM and EDX analyses confirmed comparable dentinal surface characteristics and elemental composition across all groups before demineralisation. Following pH-cycling, all specimens demonstrated evident demineralisation, characterised by increased dentinal tubule exposure on SEM and a marked reduction in mineral content on EDX analysis.

### SEM evaluation

**Figure 2:**
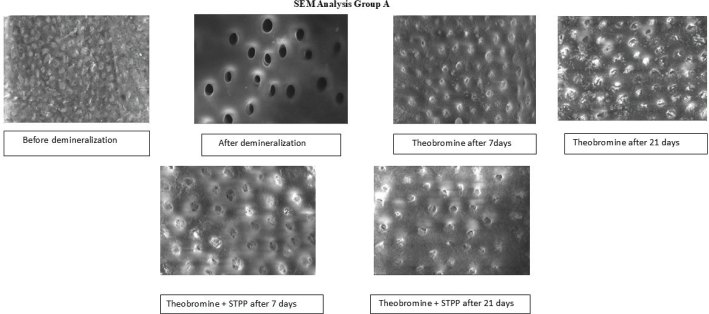
Results of SEM analysis in Group A. SEM: scanning electron microscopy.

Dentinal tubule occlusion was assessed qualitatively using SEM images and described as complete, partial or minimal occlusion. SEM analysis of Group A revealed pronounced surface demineralisation with widened and clearly exposed dentinal tubules following acid treatment. After application of theobromine for 7 days, partial tubule occlusion and initial mineral deposits were evident. By 21 days, there was greater surface uniformity, accompanied by significant tubular obliteration, indicating progressive remineralisation and surface repair (Figure 2).

**Figure 3:**
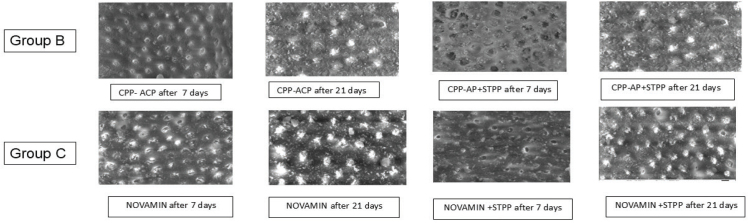
Results of SEM analysis in Groups B and C. SEM: scanning electron microscopy.

SEM evaluation of Group B demonstrated partial dentinal tubule occlusion after 7 days of CPP-ACP application, with visible mineral precipitates on the surface. At 21 days, increased crystalline deposition and enhanced tubular narrowing were observed. The combination of CPP-ACP with STPP showed comparatively greater surface coverage and more uniform tubular occlusion at both 7 and 21 days, indicating improved remineralisation efficacy over time (Figure 3).

In Group C, the Novamin application exhibited evident mineral deposition and partial tubule occlusion at 7 days. By 21 days, a denser and more homogeneous mineralised layer was noted with substantial tubular blockage. The addition of STPP enhanced surface smoothness and tubular obliteration, particularly at 21 days, suggesting synergistic remineralisation potential (Figure 3).

**Figure 4:**
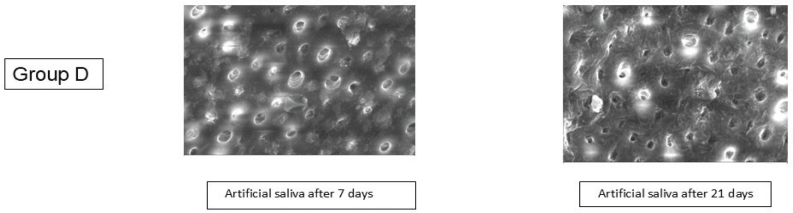
Results of SEM analysis in Group D. SEM: scanning electron microscopy.

SEM analysis of Group D (artificial saliva) showed minimal surface changes at both 7 and 21 days. The dentinal tubules remained largely open with only slight superficial deposits. No significant tubular occlusion or uniform mineral layer formation was observed, indicating limited remineralisation potential compared to the experimental groups (Figure 4 and Table 1).

**Table 1:** Comparison of SEM tubule number (before demineralisation) and after demineralisation in different groups at 0 day, day 7 and day 21.

Group	Before demineralisation	After demineralisation 0 Day	Day 7	Day 21
TB	98.39 ± 1.12	9.64 ± 1.24	71.50 ± 7.28	81.35 ± 6.19
TB+STPP	98.27 ± 1.17	8.04 ± 1.53	83.02 ± 4.18	84.81 ± 3.25
CPPACP	98.55 ± 0.97	8.75 ± 1.25	85.82 ± 3.38	87.56 ± 5.60
CPPACP+STPP	98.46 ± 0.52	6.27 ± 1.00	92.66 ± 3.74	92.49 ± 3.03
NOVAMIN	98.28 ± 1.06	7.71 ± 1.49	88.81 ± 2.53	85.68 ± 7.79
NOVAMIN+STPP	98.80 ± 0.87	12.23 ± 2.05	90.35 ± 2.87	85.98 ± 8.31
A S	99.18 ± 0.82	9.32 ± 0.84	27.91 ± 6.35	39.73 ± 8.03

Values presented for descriptive reference only; no inferential statistics applied. SEM: scanning electron microscopy.

### Percentage remineralisation (PK%)

**Figure 5:**
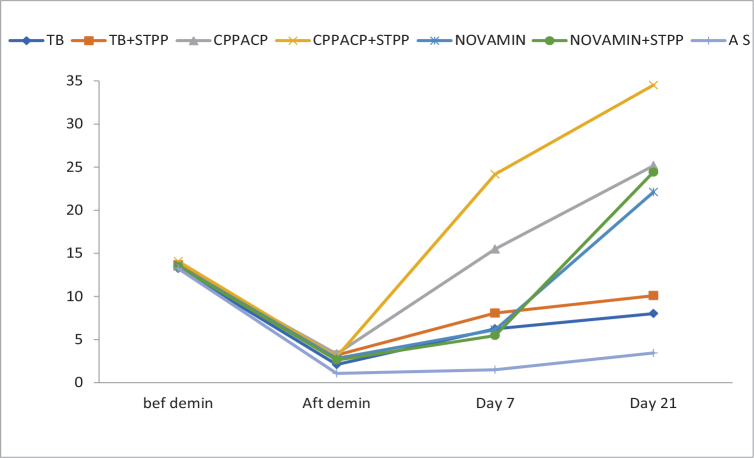
Percentage change in P-K ratio. Note: Baseline values shown for descriptive reference only.

All experimental groups demonstrated a progressive increase in percentage remineralisation from day 7 to day 21. Among the non-STPP subgroups, CPP-ACP (B1) showed greater remineralisation compared to theobromine (A1) and Novamin (C1). STPP-pretreated subgroups exhibited significantly higher remineralisation values than their corresponding non-STPP counterparts at both time points. The highest PK% was observed in the CPP-ACP with STPP group (B2) at day 21. The artificial saliva group showed negligible remineralisation throughout the experimental duration (Table 2 and Figure 5).

**Table 2:** Comparison of percentage remineralisation (PK%) among the seven groups (A1, A2, B1, B2, C1, C2 and D) across four-time intervals: baseline (before demineralisation), after demineralisation, day 7 and day 21.

Group	Before demineralisation	0 Day	Day 7	Day 21
TB	98.39 ± 1.12^a^	9.64 ± 1.24^ab^	71.50 ± 7.28^b^	81.35 ± 6.19^b^
TB+STPP	98.27 ± 1.17^a^	8.04 ± 1.53^ab^	83.02 ± 4.18^bc^	84.81 ± 3.25^b^
CPPACP	98.55 ± 0.97^a^	8.75 ± 1.25^ab^	85.82 ± 3.38^c^	87.56 ± 5.60^b^
CPPACP+STPP	98.46 ± 0.52^a^	6.27 ± 1.00^a^	92.66 ± 3.74^d^	92.49 ± 3.03^b^
NOVAMIN	98.28 ± 1.06^a^	7.71 ± 1.49^b^	88.81 ± 2.53^cd^	85.68 ± 7.80^b^
NOVAMIN+STPP	98.80 ± 0.87^a^	12.23 ± 2.05^c^	90.35 ± 2.87^cd^	85.98 ± 8.32^b^
A S	99.18 ± 0.82^a^	9.32 ± 0.84^b^	27.91 ± 6.35^a^	39.73 ± 8.03^a^
***F*-value**	**1.172**	**17.916**	**240.904**	**79.111**
***p*-value**	**0.333**	**< 0.001**	**< 0.001**	**< 0.001**

**Test done:** One-way ANOVA followed by post-hoc Tukey. Superscripts (a–d) indicate Tukey’s post-hoc homogeneous subsets. Groups sharing the same letter are not significantly different, whereas groups with different letters differ significantly at *p* < 0.05. SEM: scanning electron microscopy.

### EDX analysis

**Figure 6:**
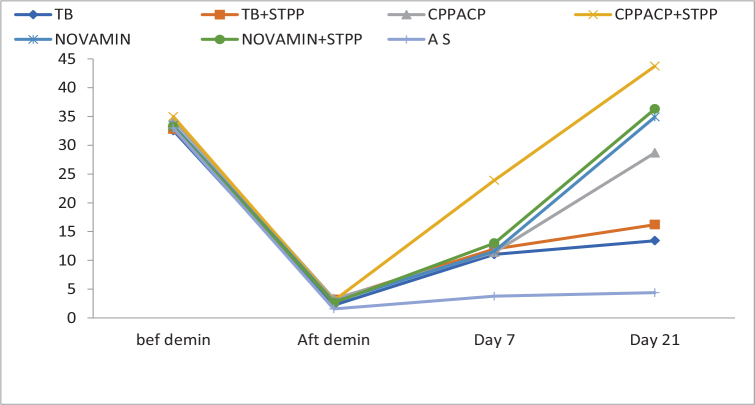
Percentage change in Ca-P ratio. Note: Baseline values shown for descriptive reference only.

EDX evaluation demonstrated a significant reduction in Ca/P ratios immediately after demineralisation in all groups. Following remineralisation, experimental groups showed a gradual increase in Ca/P values over time. At day 21, CPP-ACP with STPP (B2) exhibited Ca/P values exceeding baseline levels, indicating enhanced mineral deposition. Novamin with STPP (C2) and theobromine with STPP (A2) also showed higher Ca/P ratios compared to their respective non-STPP groups. The artificial saliva group demonstrated the lowest Ca/P values at all post-demineralisation time points (Table 3 and Figure 6). One-way ANOVA revealed statistically significant differences among groups at day 7 and day 21 (*p* < 0.05). Post-hoc analysis confirmed that STPP-pretreated subgroups showed significantly greater remineralisation and mineral gain compared to non-STPP groups and the control group.

**Table 3:** Intergroup comparison of Ca/K percentage (%) at baseline (before demineralisation), immediately after demineralisation (0 day), day 7 and day 21.

Group	Before demineralisation	After demineralisation	Day 7	Day 21
TB	32.56 ± 2.73^a^	2.20 ± 0.75^ab^	11.04 ± 1.33^b^	13.41 ± 1.43^b^
TB+STPP	32.81 ± 2.55^a^	3.20 ± 0.87^b^	11.96 ± 1.94^b^	16.20 ± 2.71^b^
CPPACP	34.38 ± 1.57^a^	3.34 ± 1.03^b^	11.41 ± 1.73^b^	28.68 ± 5.52^c^
CPPACP+STPP	34.98 ± 2.39^a^	2.99 ± 0.40^b^	23.91 ± 2.40^c^	43.73 ± 3.19^e^
NOVAMIN	33.48 ± 2.19^a^	2.82 ± 1.48^b^	11.58 ± 1.70^b^	34.90 ± 2.88^d^
NOVAMIN+STPP	33.18 ± 1.95^a^	2.63 ± 1.09^b^	12.99 ± 1.53^b^	36.29 ± 2.41^d^
A S	32.97 ± 1.03^a^	1.56 ± 0.76^a^	3.77 ± 0.41^a^	4.39 ± 0.27^a^
***F*-value**	1.725	4.150	124.962	222.528
***p*-value**	0.130	0.001	< 0.001	< 0.001

**Test done:** One-way ANOVA followed by post-hoc Tukey. Superscript letters (a–e): Within each time interval, letters represent Tukey’s Honest Significant Difference (HSD) homogeneous subsets. Means that share the same superscript letter are **not** significantly different from each other (*p* ≥ 0.05). Means with different letters are significantly different (*p* < 0.05).

## Discussion

The present study evaluated the remineralisation potential of theobromine, CPP-ACP and Novamin, with and without STPP pretreatment, on artificially demineralised dentine using SEM and EDX analyses. The results demonstrated that dentine remineralisation is a time-dependent process and is significantly enhanced by biomimetic phosphate-assisted strategies.

Dentine demineralisation begins at a higher critical pH (6–6.9) compared with enamel due to its lower mineral content and higher organic matrix composition [4]. Non-carious lesions such as abrasion, erosion, attrition, abfraction and fractures expose dentine to the oral environment, making it susceptible to acid-mediated mineral loss [7, 32]. The present in vitro model using pH cycling effectively simulated these conditions and produced consistent artificial caries-like lesions in dentine (Tables 1–3) [2, 33–40].

NCPs such as Dentin sialophosphoprotein (DSPP) and Dentin Matrix Acidic Phosphoprotein 1 (DMP1) naturally mediate this process in vivo; STPP effectively mimics their function by providing phosphate groups for nucleation [19, 41–43]. SEM analysis (Table 1) revealed significant occlusion of dentinal tubules after remineralisation in all experimental groups. The groups pretreated with STPP (A2, B2, C2) exhibited greater tubule occlusion and more homogeneous mineral deposition than their non-STPP counterparts. This can be explained by the biomimetic remineralisation concept, in which STPP acts as a phosphorylating agent, inducing pre-nucleation sites on collagen fibrils and facilitating intrafibrillar hydroxyapatite deposition [18, 44].

The quantitative evaluation using PK% (Table 2) showed that CPP-ACP + STPP (B2) achieved the highest remineralisation after 21 days, followed by Novamin + STPP (C2) and theobromine + STPP (A2). CPP-ACP provides stabilised ACP clusters, maintaining a supersaturated calcium and phosphate environment, which facilitates both intra- and inter-tubular mineral deposition [26–28]. Novamin, a bioactive glass, releases calcium and phosphate ions in the presence of saliva, promoting surface hydroxycarbonate apatite formation [29–31]. Theobromine, although less potent than CPP-ACP or Novamin, demonstrated moderate remineralising potential by enhancing hydroxyapatite crystallite growth [32, 33].

EDX elemental analysis (Table 3) supported SEM and PK% findings, showing significant increases in Ca/P in all treatment groups over 21 days. STPP-treated groups consistently exhibited higher mineral content compared with non-STPP groups, confirming the role of phosphate-mediated collagen phosphorylation in promoting biomimetic mineralisation [18, 44]. Artificial saliva alone (Group D) showed minimal remineralisation, highlighting the necessity of active remineralising agents for restoring dentine mineral content [38, 39].

Time-dependent evaluation indicated that the majority of mineral deposition occurred between day 7 and day 21, confirming previous findings that a minimum of 2–3 weeks is required for effective in vitro dentine remineralisation under simulated oral conditions [28]. The combined use of STPP and remineralising agents resulted in more uniform intrafibrillar and intertubular mineral deposition, which is crucial for restoring dentine mechanical properties and reducing hypersensitivity [18, 41, 44].

Overall, the results corroborate the concept that biomimetic remineralisation strategies, particularly CPP-ACP and STPP, can effectively restore demineralised dentine. The findings also highlight the potential clinical relevance of these agents in managing early non-carious and carious lesions, reducing the risk of progression and providing a foundation for minimally invasive restorative approaches [45–50].

Limitations of this study include its in vitro nature, absence of pulpal pressure simulation, lack of mechanical property assessment, short observation period and prolonged STPP pretreatment time, which may limit direct clinical translation. Future studies should evaluate bond strength, nano-indentation and in situ or clinical performance of STPP-based biomimetic systems.

## Conclusion

Within the limitations of this in vitro study, among the tested agents, CPP-ACP and Novamin achieved substantial remineralisation within 7 days in the presence of STPP, whereas theobromine showed slower remineralisation and may require a longer duration to achieve comparable mineral deposition. These findings suggest that phosphate-assisted biomimetic strategies can effectively restore demineralised dentine and may serve as a basis for minimally invasive clinical interventions.

## Data Availability

The data that support the findings of this study are available from the corresponding author upon reasonable request.
